# Association between Air Pollution and General Outpatient Clinic Consultations for Upper Respiratory Tract Infections in Hong Kong

**DOI:** 10.1371/journal.pone.0086913

**Published:** 2014-01-23

**Authors:** Wilson W. S. Tam, Tze Wai Wong, Lorna Ng, Samuel Y. S. Wong, Kenny K. L. Kung, Andromeda H. S. Wong

**Affiliations:** 1 Jockey Club School of Public Health and Primary Care, The Chinese University of Hong Kong, Sha Tin, Hong Kong; 2 Shenzhen Municipal Key Laboratory for Health Risk Analysis, Shenzhen Research Institute of The Chinese University of Hong Kong, Shenzhen, Guangdong Province, China; 3 General Outpatient Clinics, Kwong Wah Hospital, Yaumatei, Hong Kong; The Ohio State University, United States of America

## Abstract

**Background and Objectives:**

Many studies have shown the adverse effects of air pollution on respiratory health, but few have examined the effects of air pollution on service utilisation in the primary care setting. The aim of this study was to examine the association between air pollution and the daily number of consultations due to upper respiratory tract infections (URTIs) in general outpatient clinics (GOPCs) in Hong Kong.

**Methods:**

Daily data on the numbers of consultations due to URTIs in GOPCs, the concentrations of major air pollutants, and the mean values of metrological variables were retrospectively collected over a 3-year period (2008–2010, inclusive). Generalised additive models were constructed to examine the association between air pollution and the daily number of consultations, and to derive the relative risks and 95% confidence intervals (95% CI) of GOPC consultations for a unit increase in the concentrations of air pollutants.

**Results:**

The mean daily consultations due to URTIs in GOPCs ranged from 68.4 to 253.0 over the study period. The summary relative risks (and 95% CI) of daily consultations in all GOPCs for the air pollutants PM_10_, NO_2_, O_3_, and SO_2_ were 1.005 (1.002, 1.009), 1.010 (1.006, 1.013), 1.009 (1.006, 1.012), and 1.004 (1.000, 1.008) respectively, per 10 µg/m^3^ increase in the concentration of each pollutant.

**Conclusion:**

Significant associations were found between the daily number of consultations due to URTIs in GOPCs and the concentrations of air pollutants, implying that air pollution incurs a substantial morbidity and increases the burden of primary health care services.

## Introduction

Most epidemiological time series studies on air pollution focus on hospital admissions and mortality as health outcomes. [1]–[6] These outcomes represent, respectively, serious morbidity and the ultimate health consequence of air pollution. Illnesses seen in primary health care settings, by contrast, form the much wider base of the ‘pyramid’ of air pollution-related diseases, but are much less studied.

Respiratory diseases are very common in all age groups and generate a major demand on health care services worldwide. Acute respiratory infections represent one of the most common reasons for seeking medical attention in the primary health care setting. [7] A recent cross-sectional morbidity study in Hong Kong revealed that 26.4% of outpatient consultations were due to upper respiratory tract infections [8].

Gaseous air pollutants such as sulphur dioxide (SO_2_), ozone (O_3_), and nitrogen dioxide (NO_2_) have been shown to cause irritation and constriction of the large airways. [9] However, only a few studies have examined the association between air pollution and the daily number of consultations in the primary health care sector. [10]–[13] One reason was the lack, in most countries, of routinely collected data on primary care service utilisation in both private and public sectors; hence, data might need to be collected prospectively. For example, in a previous study we conducted in Hong Kong, we invited 13 private general practitioners (GPs) to manually record the daily number of patients consulted for respiratory diseases. We used these data to examine the association between the number of GP consultations and the concentration of air pollutants [13].

In Hong Kong, primary health care is provided by both the private general practitioner clinics and the public general out-patient clinics (GOPCs). The GOPCs network covers all districts in Hong Kong, and provides primary care services mainly for the socially disadvantaged, while middle- to high-income groups often prefer private GP clinics for their primary care needs. Proportionately, more elderly patients and those with chronic diseases attend the GOPCs, whereas more patients with acute health problems consult GPs in private clinics. The GOPCs are heavily subsidised by the government; the charge per consultation in these clinics (HK$45 or ∼US$6) is around a quarter of the median charges in private clinics, which range from US$19 to US$26. [14] The Hospital Authority (HA), a Hong Kong government-funded ‘statutory board’, manages the public GOPC network, and since 1999, medical records in these clinics have progressively been computerised [15].

The aim of this study is to examine the effects of air pollution on the frequency of consultations for upper respiratory tract infections (URTIs) in the public primary care clinics. We retrospectively collected data on daily consultations due to URTIs, at the GOPCs located in two of seven geographically-defined hospital and clinic ‘clusters’ under the Hospital Authority, from 2008 to 2010. We used a time-series approach to investigate the association between air pollutant concentrations and GOPCs consultations.

## Materials and Methods

### Data

**Figure 1:**
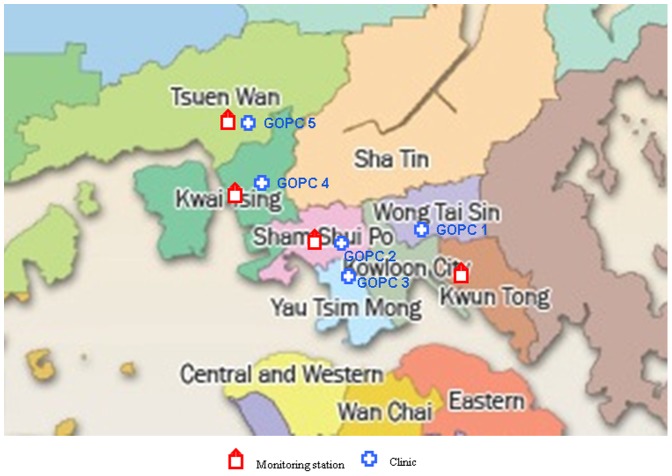
A location map of GOPCs and the air monitoring stations.

This is a retrospective time series study. We identified ‘upper respiratory tract infection’ by the diagnostic code R74, defined as ‘URI (head cold)/rhinitis, not otherwise classified’ in the Revised Edition of the International Classification of Primary Care 2 (ICPC-2), prepared by the World Organization of National Colleges, Academies, and Academic Associations of General Practitioners/Family Physicians (WONCA). [16] Under this classification system, R74 excludes specific diagnostic labels such as acute and chronic sinusitis (R75), acute tonsillitis (R76), acute laryngitis/tracheitis/croup (R77), acute bronchitis/bronchiolitis (R78), and influenza (R80). Data on the number of daily visits for URTIs (R74) were extracted from the Clinical Data Analysis & Reporting System of the Hospital Authority, for five of the HA’s GOPCs. Three of the GOPCs were in the Kowloon Central Cluster, which covers the Kowloon peninsula – a flat, densely populated urban area. The other two, part of the Kowloon West Cluster, were located in the north-western ‘new towns’ of Kwai Tsing and Tsuen Wan which, besides being residential, also have a container terminal and an industrial estate, respectively. Data were retrospectively collected from 1 January 2008 to 31 December 2010. [15] Four monitoring stations of the Hong Kong Government’s Environmental Protection Department were matched to the five GOPCs according to their geographic locations; one of the monitoring stations served both GOPC 2 and GOPC 3 (Figure 1).

Four pollutants were regularly monitored in all of the monitoring stations. These were: nitrogen dioxide (NO_2_), sulphur dioxide (SO_2_), ozone (O_3_), and particulates with an aerodynamic diameter less than 10 µm (PM_10_). Data regarding hourly concentration of these pollutants were obtained for the corresponding 3-year period as the clinical data (2008–2010) from the Environmental Protection Department (www.epd.gov.hk) and the collection method has been described in our previous study. [13] The daily mean concentration of each pollutant was computed as the average of the hourly data, if more than two-thirds of the hourly data were available for that day; otherwise, the daily mean was deemed as missing. Missing daily concentrations in one station were predicted by a regression of data from that station on the corresponding data from the nearest neighbouring station. [17] The daily mean temperatures and relative humidity at different districts were acquired from the Hong Kong Observatory (www.hko.gov.hk).

### Statistical Modelling

A generalised additive model, using the Poisson distribution with a log-link function, was used to construct a core model for each of the five GOPCs. [18] In brief, each model regressed the daily numbers of the URTIs consultations on several variables: time (day), day of the week, daily mean temperature and humidity, and a holiday indicator. To adjust for the potential confounding effect of influenza on the number of URTIs consultations, we used a dichotomous variable to indicate the weeks during which the number of influenza consultations exceeded the 75^th^ percentile for the study period. This method has been used in time series studies of air pollution and hospital admissions for respiratory illnesses. [19] The estimated weekly numbers of influenza were obtained from the Centre for Health Protection, Hong Kong SAR Government (www.chp.gov.hk). We used penalised smoothing splines [20], [21] to adjust for seasonal patterns and long-term trends in daily consultations, temperature, and relative humidity, with degrees of freedom (df) selected a priori, based on previous studies. [22], [23] Specifically, we used 7 df per year for time trends, 6 df for temperature and 3 df for humidity [19].

The quasi-likelihood method was used to correct for over-dispersion. [18] To minimise autocorrelation, which would bias the standard errors, we specified that the absolute values of the partial autocorrelation function for the model residuals had to be <0.1 for the first 2 lag days. [24] When these criteria were not met, we added autoregressive terms for the outcome variable to the core model, up to a maximum of three autoregressive terms.

Linear effects of all the air pollutants for the same day (lag 0) up to 3 lag days (lag 3) were tested in each model and the best lag for each pollutant at the GOPC was chosen as the one that yielded the largest t-value.

We conducted all analyses using the MGCV package in R [25] and expressed the results as the relative risks (RRs) of visits for URTIs in each GOPC for every 10 µg/m^3^ increase in the concentrations of all four pollutants. The best lag RRs for each pollutant, obtained from individual GOPCs, were then combined using random effects models. [26] Ethics approval was obtained for conducting this study from the Research Ethics Committee, Kowloon West Cluster, Hospital Authority (Ref: KW/EX-11–137). Our study did not involve any patient’s personal information.

## Results

### Daily Consultations at GOPC

Table 1 shows summary statistics of the consultations due to URTIs, at the five GOPCs, over the study period. From 2008 to 2010, the total number of consultations was 817,240, with mean daily consultations ranging from 68.4 to 253.0. The district-specific demographic information for each GOPC is also shown.

**Table 1:** Daily number of URTIs consultations at the five GOPCs, and district specific statistics, from 1 Jan 2008 to 31 Dec 2010.

	GOPC 1	GOPC 2	GOPC 3	GOPC 4	GOPC 5
Mean (SD)	179.5 (97.3)	115.8 (70.4)	68.4 (48.6)	253.0 (149.2)	130.1 (68.5)
Median	197	132	78	288	136
Quartile	99, 253	68, 165	24, 107	163, 360	71.5, 175
District specific statistics *					
Area (km^2^)	9.36	9.48	6.85	21.82	60.70
Population	420,183	380,855	307,878	511,167	304,637
Mean age (years)	44.5	43.2	41.1	41.9	41.3
Male gender	46.7%	46.7%	46.4%	47.3%	46.6%
Never married**	33.7%	31.2%	29.5%	32.6%	28.9%
Post- secondary educated or above***	17.9%	22.8%	31.8%	18.7%	29.8%
Mean number of persons in household	2.9	2.7	2.7	3.0	2.9
Mean household income	$17,000	$16,280	$22,070	$17,000	$24,100

* Information extracted from 2011 Population Census by the Census of Statistics Department, Hong Kong Special Administrative Region Government. The two districts served by GOPC 4 and 5 are much larger than the others, as they include uninhabited country park areas. The populations in these districts are concentrated in urban areas, much like the other districts.

** aged 15 or above.

*** non-student population aged 20 or above.

### Descriptive Statistics for Air Pollutants

Table 2 shows the mean, median, and quartile values of the daily air pollutant concentrations measured at each monitoring station throughout the study. During this period, the mean daily temperature and humidity in Hong Kong were 23.3°C (SD = 5.2) and 78.1% (SD = 11.1) respectively. Table 3 shows the Pearson’s correlations between the air pollutants, listed by each monitoring station.

**Table 2:** Summary statistics of the four air pollutants in the four monitoring stations from 1 January 2008 to 31 December 2010.

Station	Pollutant	Mean (SD) (µg/m^3^)	Median (µg/m^3^)	Quartile (µg/m^3^)
Station 1 for GOPC 5	NO_2_	62.7 (20.1)	58.5	48.2, 73.2
(Missing: 39 days)	PM_10_	48.7 (28.9)	41.9	28.8, 62.9
	O_3_	30.8 (19.6)	26.4	14.1, 43.8
	SO_2_	19.8 (13.5)	17.2	10.7, 25.1
Station 2 for GOPC 4	NO_2_	64.9 (21.8)	59.7	49.6, 75.7
(Missing: 44 days)	PM_10_	47.8 (27.4)	42.0	29.3, 62.1
	O_3_	30.5 (20.3)	27.2	13.2, 43.6
	SO_2_	23.6 (19.9)	15.3	8.1, 36.9
Station 3 for GOPC 2 & 3	NO_2_	67.5 (22.0)	66.6	50.8, 79.7
(Missing: 23 days)	PM_10_	49.1 (29.9)	42.8	27.9, 64.0
	O_3_	28.3 (19.1)	23.6	12.8, 40.0
	SO_2_	16.6 (14.4)	11.9	7.3, 20.8
Station 4 for GOPC 1	NO_2_	60.8 (20.3)	56.2	47.1, 70.9
(Missing: 43 days)	PM_10_	49.0 (31.8)	42.6	28.9, 63.9
	O_3_	33.4 (21.9)	30.5	14.0, 48.5
	SO_2_	16.4 (16.3)	10.3	6.9, 17.8

Remark: ‘Missing’ indicates that at least one pollutant measurement was missing that day.

**Table 3:** Correlation between pollutants by monitoring station.

Station	Pollutant	NO_2_	PM_10_	O_3_	SO_2_
Station 1 for GOPC 5	NO_2_	1	0.58**	0.24**	0.37**
(Missing: 39 days)	PM_10_		1	0.45**	0.21**
	O_3_			1	−0.22**
	SO_2_				1
Station 2 for GOPC 4	NO_2_	1	0.60**	0.14**	0.27**
(Missing: 44 days)	PM_10_		1	0.39*	0.06*
	O_3_			1	−0.46*
	SO_2_				1
Station 3 forGOPC 2 & 3	NO_2_	1	0.61**	0.34**	0.38**
(Missing: 23 days)	PM_10_		1	0.46**	0.26**
	O_3_			1	−0.07*
	SO_2_				1
Station 4 for GOPC 1	NO_2_	1	0.50**	0.14**	0.36**
(Missing: 43 days)	PM_10_		1	0.42**	0.15**
	O_3_			1	−0.22**
	SO_2_				1

* p<0.05;

** p<0.01.

### Relative Risk of Daily Consultation

The individual relative risks (RRs) of consultation due to URTIs by GOPC, and the summary RRs for all GOPCs (per 10 µg/m^3^ increase in the concentrations of each air pollutant) are shown in Table 4. The RRs for NO_2_ in individual clinics were consistently significant. For PM_10_, the RRs were significant in three out of five clinics while the RRs for O_3_ were significant in four out of five clinics. For SO_2_, none of the RRs in individual clinics were significant, although all were above unity. Statistically significant summary RRs were found for NO_2_, PM_10_, and O_3_, while the combined RR for SO_2_ was marginally significant (p = 0.060). The summary relative risks (with 95% confidence intervals) from all GOPC for NO_2_, PM_10_, O_3_, and SO_2_ were 1.005 (1.002, 1.009), 1.010 (1.006, 1.013), 1.009 (1.006, 1.012) and 1.004 (1.000, 1.008) respectively, per 10 µg/m^3^ increase in the concentration of each air pollutant.

**Table 4:** Relative risks and 95% CI of the visits to five GOPCs for upper respiratory tract infections (URTIs), per 10 µg/m^3^ increase in the concentration of air pollutants.

Clinic	NO_2_	PM_10_	O_3_	SO_2_
GOPC 1	1.007 [lag 0 day]	1.007 [lag 1 day]	1.010 [lag 1 day]	1.001 [lag 0 day]
	(1.000, 1.013)*	(1.004, 1.010)*	(1.004, 1.016)*	(0.992, 1.009)
GOPC 2	1.014 [lag 0 day]	1.005 [lag 0 day]	1.003 [lag 1 day]	1.014 [lag 0 day]
	(1.000, 1.029)*	(0.998, 1.013)	(0.988, 1.018)	(0.998, 1.031)
GOPC 3	1.013 [lag 0 day]	1.011 [lag 3 days]	1.015 [lag 1 day]	1.010 [lag 3 days]
	(1.004, 1.023)*	(1.006, 1.017)*	(1.004, 1.025)*	(0.999, 1.022)
GOPC 4	1.010 [lag 3 days]	1.005 [lag 3 days]	1.010 [lag 1 days]	1.002 [lag 0 day]
	(1.004, 1.015)*	(1.001, 1.009)*	(1.004, 1.016)*	(0.996, 1.009)
GOPC 5	1.010 [lag 0 day]	0.999 [lag 3 days]	1.010 [lag 3 day]	1.003 [lag 0 day]
	(1.002, 1.017)*	(0.995, 1.003)	(1.003, 1.018)*	(0.993, 1.014)
Combined	1.010	1.005	1.009	1.004
	(1.006, 1.013)*	(1.002, 1.009)*	(1.006, 1.012)*	(1.000, 1.008)#

* p<0.05;

# p = 0.06.

## Discussion

While air pollution has been extensively researched with regard to its association with mortality and hospital admissions, studies reporting its effects on primary care consultations are scarce.[11], [13], [27]–[30] This time series study is one of the few that investigate air pollution’s effects on primary health care service utilisation in the public sector. We found significant positive associations between three air pollutants – namely, NO_2_, O_3_, and PM_10_ (from zero to three lag days) – and the daily number of consultations for URTIs at outpatient clinics in Hong Kong.

The combined RRs for NO_2_, O_3_, and PM_10_ (at 1.010, 1.009 and 1.005 respectively) for visits to public clinics herein investigated were substantially lower than their corresponding RRs reported for private GP visits in Hong Kong (at 1.030, 1.024 and 1.020 respectively) in our previous study in 2006. [13] One possible explanation is that the GOPCs services required patients to register either by walk-in or by phone, with daily quotas placed on the number of consultations. Because of the much lower prices charged by the GOPCs compared to that charged by the private sector GPs, the waiting time for patients attending GOPCs could be much longer than that for the latter. [31] Therefore, it is plausible that to reduce waiting time, patients with more severe URTIs may have consulted private GPs or even the accident and emergency departments of hospitals. This spillage of patients into alternative services could then result in the smaller RRs found at the GOPCs in this study. This would explain the findings in this study that the ‘response’ or ‘health outcome’ to a unit increase in the concentration of air pollutants, as GOPCs visits, was lower than consultations to private GPs. The lack of a significant association with SO_2_ could be explained by the relatively low concentrations of SO_2_ in Hong Kong. Levels of SO_2_ in three of the four stations were within the air quality guidelines of 20 µg/m^3^ recommended by the World Health Organization. [9] By contrast, the mean concentrations of NO_2_ and PM_10_ in all stations were much higher than their annual air quality guidelines,

The lag times in both Hong Kong studies were similar, ranging from zero to three lag days. In a GP study in London, significant RRs for SO_2_ and PM_10_ were reported for specific diseases (allergic rhinitis and asthma) and in different age groups for URTIs, as the 10^th^ to the 90^th^ percentile change in air pollutant concentrations, from zero to 3 lag days. [27], [28] This makes comparison of RRs difficult. Ostro reported similar associations for PM_10_ and O_3_ with URTIs or lower respiratory tract infections (LRTIs) among children of different age groups in Santiago, Chile. [29] In an ambulatory care study in Atlanta, Georgia, in the USA, Sinclair and Tolsma found weak but significant associations between PM and consultations for asthma and LRTIs. They reported a longer lag time of 3 to 5 days. [30] Differences in lag days may have been due to differences in the primary health care settings and in consultation behaviour across different cities.

Besides time series studies, spatial studies have also reported the association between air pollution and primary care utilisation. Hwang et al. reported that carbon monoxide (CO), NO_2_, SO_2_, and PM_10_ all had significant effects on daily visits to clinics due to LRTIs, based on small area design and hierarchical modelling of data from 50 communities in Taiwan. [12] Oiamo et al. used a community health survey on GP access and utilisation in Sarnia, Ontario, in Canada, and data on spatial differences in air pollutant concentrations, to demonstrate their relationships. [32] A time series study that took place over the 2008 Olympic Games in Beijing showed a significant reduction in outpatient visits for asthma during the air pollution control period, during which traffic and industrial emissions were restricted. [33] This provided strong evidence of a cause-effect relationship between air pollution and respiratory illnesses in an outpatient setting.

Ciencewicki and Jaspers have suggested a potential mechanism linking exposure to environmental air pollutants to their adverse health effects – including those effects related to respiratory infections. Pollutants could induce oxidative stress, resulting in the production of free radicals. These, in turn, could have damaging effects on the respiratory system, thereby lowering the resistance of the tissues to viral and bacterial infections. [34] Also, the oxidative stress induced by exposure to air pollutants may enhance the morbidity of an infection through an increased inflammatory response. Potential mechanisms of individual pollutants have been reported elsewhere [35]–[38].

A major strength of our study is the large number of consultations (which increases the study’s power) and the reliability of the data sources, as all GOPCs data of the Hospital Authority are stored in one computerized database. In addition, we have a comprehensive network of air quality monitoring stations in different districts of the city. There are several limitations to our study results. First, as our study area was limited to one region of the city, and our GOPCs visitors belonged to the lower social class, selection bias is a possibility. Secondly, misclassification of the respiratory infections is a potential problem. For instance, in an outpatient setting, some LRTIs such as acute bronchitis or influenza might be wrongly coded as URTIs. This would have resulted in wider confidence intervals of the RRs.

Upper respiratory tract infections should include R74 to R77 in the ICPC-2 codes. Owing to logistical constraints in the acquisition of daily data, we were only able to download one diagnostic code from the system. A check on the annual data (which could be accessed more easily) showed that the total number of cases coded as R75–77 was about 1% of the cases coded as R74. The mean daily number of the former group ranged from 0.9 to 2,6 cases in each of the five clinics, whereas the corresponding mean daily number of cases coded as R74 ranged from 68–253. Hence, we believe that our findings would not deviate much if we had included all the URTI codes.

Furthermore, all patients attending one clinic were assigned the same level of exposure to air pollutants according to data from the nearest monitoring station. In reality, individual exposure could be quite different from the ambient pollution concentration. We cannot rule out the possibility that some patients living in one district would use GOPCs in another district, because the distances between some clinics are fairly short. Hence, as in other ecological studies, the misclassification of patients’ true exposure to air pollution is a potential source of error that is difficult to assess. Nonetheless, the data on air pollutants show little variation between districts (Table 2), as our study areas were adjacent to each other, and consisted largely of flat terrain. In addition, the possible biases discussed above may be assumed as about constant over the study period, and therefore they should not have remarkably confounded the positive relationship found between changes in the concentration of air pollutants and the GOPCs visits for URTIs.

In our choice of health outcomes, we only focused on URTIs, the only illnesses with a sufficiently large number of mean daily consultations for time series analysis. By contrast, the average daily number of visits for asthma in our clinics ranged from 6 to 11. Many existing studies focused on PM_2.5_, which have more damaging effects on the lungs. However, it was not routinely measured in all monitoring stations. Moreover, PM_10_ are more relevant because of their effects on the upper respiratory tract.

## Conclusion

Our results showed a significant association between the concentrations of several air pollutants and the daily number of GOPCs consultations due to URTIs in Hong Kong. These findings provide further evidence that air pollution is a major public health problem. The number of consultations in primary care represents much higher than hospital admissions and deaths. Air pollution thus incurs a substantial burden to health care services in an urban community.
